# Magnesium and Other Biometals in Oxidative Medicine and Redox Biology

**DOI:** 10.1155/2017/7428796

**Published:** 2017-10-04

**Authors:** Martin Kolisek, Rhian M. Touyz, Andrea Romani, Mario Barbagallo

**Affiliations:** ^1^Comenius University in Bratislava, Jessenius Medical Faculty in Martin, Biomedical Center Martin, Martin 03601, Slovakia; ^2^University of Glasgow, Glasgow G12 8TA, UK; ^3^Case Western Reserve University, Cleveland, OH 44116-4970, USA; ^4^University of Palermo, Palermo 90144, Italy

Magnesium (Mg) and also other biometals (BM) play an essential role in regulating a plethora of biological processes such as (1) metabolism of biomacromolecules, (2) cell division and proliferation, (3) normal mitochondrial homeostasis and energy production, (4) normal redox homeostasis, (5) antioxidant signaling, and (6) crosstalk between major signal cascades. However, exact molecular mechanisms underlying the action of Mg or other BM in these processes are often only partially understood. This may relate, in part, to lack of sensitive and specific tools to assess Mg and BM in biological systems and to relative lack of emphasis of Mg and BM in clinical medicine.

*Diabetes mellitus* type 2 (DT2) might serve as an example. Hypomagnesaemia has been identified in 9 to 40% of DT2 patients in “Mg-focused” clinical trials. However, Mg status in DT2 patients is rarely determined as routine clinical practice.

Another example highlighting the importance of Mg homeostasis in disease etiology is Parkinson disease (PD). It is not yet clear whether chronic intracellular Mg deficiency causes the disease itself but it is obvious that insufficient dietary intake of Mg or its wasting (GIT, kidney) worsens PD symptoms and accelerates its progression.

Manganese (Mn) is an essential trace element involved in many physiological processes supporting growth and development, and also neuronal functions. On the other hand, pathological accumulation of Mn in the brain has a detrimental, toxic effect on neurons. Dopaminergic neurons in the *substantia nigra* are especially sensitive to Mn toxicity; thus, accumulated Mn may cause manganism, a disease condition with etiology almost identical with PD.

Even though copper (Cu) plays a role in multiple vital enzymatic reactions and physiological processes, it is notoriously known for its essential role in redox homeostasis in cells and consequently tissues and organs of the body. For instance, decreased levels of protein-bound Cu may lead to iron (Fe) accumulation in the brain, thus increased oxidative stress (OS) that is hallmarking prevalence and progression of neurodegenerative and psychiatric diseases. The two pathological conditions resulting directly from the perturbed transport of Cu in the body are Menkes disease (negatively affected is the intestinal P-type ATPase ATP7A transporting Cu^+^) and Wilson's disease (negatively affected is the P-type ATPase ATP7B transporting Cu^+^ that is localized within trans-Golgi network of hepatocytes and brain cells).

BM not only are important for global biochemistry and physiology of the body, but also have been popular in the field of implantology. For example, low toxicity, durability (when in alloys), and biodegradability made out of Mg a “super-component” of materials that are used for manufacturing of the latest generation of stents or other biodegradable implants. The field of implantology and implant material engineering is progressing rapidly, and it is likely that the success of Mg will be followed by other BM soon.

Processes maintaining normal mitochondrial homeostasis (MH) are essential for life and involve reactive oxygen species (ROS). Excess bioavailability of ROS (oxidative stress) contribute to cell dysfunction, injury, and mitophagy/autophagy. At the levels of cells and also the whole body, MH deterioration leads to senescence and death. Certain organs (especially those metabolically highly active, e.g., brain, heart, muscles, and liver) are more prone to deterioration of MH than others. Thus, natural ageing may be paralleled with premature ageing of particular organs that often demonstrates as progressive degenerative disease. The factors behind premature ageing of any organ might be encoded genetically, or they have epigenetic, or environmental background, or a combination of all. Mild OS (e.g., Akt-mediated mitochondrial OS) triggers mitophagy. Excessive, strong OS leads to death of the cells. Disbalanced homeostasis of redox-active BM such as Cu, Fe, Mn, Zn, and Mg might have deleterious effects on MH. Therefore, parameters defining status of the homeostasis of aforementioned BM should be routinely considered by the clinicians to project correctly an integrative clinical image of the patient that is necessary to adjust the most appropriate therapy.

Similar to PD, Alzheimer's disease (AD) has been linked to excessive OS, disturbed BM homeostasis, and disturbed MH. I.-M. Balmus et al. in their work assessed (1) levels of Mn, Mg, and Fe, (2) activities of superoxide dismutase and glutathione peroxidase, and (3) concentration of malondialdehyde (lipid peroxidation marker) in blood sera of healthy probands, sera of patients with mild cognitive impairment (MCI), and blood sera of patients with diagnosed AD. These authors found increased lipid peroxidation, low antioxidant defense, low Mg and Fe concentrations, and high Mn levels in MCI and AD patients, in a gradual manner. Outcomes of this study clearly demonstrate aberrant BM homeostasis with OS in MCI and AD. Moreover, these data may help to develop a predictive protocol that could complement AD biomarkers that are already being tested in large clinical trials.

Both, 3-hydroxyanthranilic acid (3-HANA) and 3-hydroxykynurenine (3-HK) are intermediates in the metabolism of tryptophan. 3-HANA was initially considered neurotoxic but later identified as having a neuroprotective effect with therapeutic potential in neuroinflamatory disorders such as AD. On the other hand, elevated levels of 3-HK are having clear neurotoxic effects linked to pathologies of AD and early stage Huntington disease (HD). D. Ramírez-Ortega et al. studied the effect of 3-HANA and 3-HK on Cu toxicity in primocultures of rat astrocytes. These authors identified both kynurenines (1) to potentiate the Cu cytotoxicity in ROS-independent manner and (2) to potentiate the effect of Cu on the decrease of glutathione (GSH) levels. Kynurenine pathway (KP) plays an important role in regulation of OS and inflammation, and in pathologies of major neurodegenerative disorders. Therefore, work of group around D. Ramírez-Ortega et al. urges for further examination of the crosstalk between metabolites of KP and homeostasis of Cu (and perhaps also homeostasis of other BM).

I. Pilchova et al. discuss in their review involvement of Mg regulation of cellular and mitochondrial functions focusing their attention primarily on energy metabolism, mitochondrial calcium (Ca^2+^) handling, and apoptosis. This work provides an up-to-date topic and emphasizes the importance of mitochondrial Mg homeostasis (MMH) beyond mitochondria and that aberrant MMH may have detrimental effects on cell. At several occasions, the importance of mitochondria-endoplasmic reticulum (ER) crosstalk, in respect to Mg homeostasis and essential intracellular processes, is being accentuated.

The central role of BM in the maintenance of oxidative balance within the frame of metabolic and neurodegenerative disorders is discussed by M. Pokusa and A. K. Trancikova. The review highlights the intersection between etiopathologies of neurodegeneration and of metabolic disorders. It also features ROS and disturbed BM homeostasis as being causative (and perhaps also consecutive) hallmarks of the aforementioned disease conditions.

As previously mentioned, Mg is also a focus of implantology and biomaterial engineering due to its low toxicity and biodegradability. Z. Liu et al. in their work highlight unique properties of Mg and microbicide effect of silver (Ag; Ag nanoparticles generate ROS in living biological systems). By controlling the microstructure and increasing the Ag content, authors obtained Mg-Ag alloys with good antibacterial properties in harsh and dynamic conditions and with almost equivalent cytocompatibility to human primary osteoblasts as pure Mg.

Papers in this special issue highlight new exciting data, comment, and synthesise the newest knowledge on Mg and other BM in oxidative medicine and redox biology. We hope that this special issue will attract broad readership in the field spanning from neurodegenerative to metabolic disorders and implantology. We would like to express our thanks to all the authors, reviewers, and the editorial team for the great support in making this special issue a reality.

*Martin Kolisek* *Rhian M. Touyz* *Andrea Romani* *Mario Barbagallo*

